# Screen time among school-aged children of aged 6–14: a systematic review

**DOI:** 10.1186/s41256-023-00297-z

**Published:** 2023-04-19

**Authors:** Jingbo Qi, Yujie Yan, Hui Yin

**Affiliations:** 1 School of Public Health, Peking University, 38 Xueyuan Road, Haidian District, Beijing, 100191 People’s Republic of China; 2 China-Japan Friendship Hospital, 2 East Yinghuayuan Street, Chaoyang District, Beijing, 100029 People’s Republic of China

**Keywords:** Screen time, School-aged children, Systematic review

## Abstract

**Background:**

Screen time refers to the time an individual spends using electronic or digital media devices such as televisions, smart phones, tablets or computers. The purpose of this study was to conduct systematic review to analyze the relevant studies on the length and use of screen time of school-aged children, in order to provide scientific basis for designing screen time interventions and perfecting the screen use guidelines for school-aged children.

**Methods:**

Screen time related studies were searched on PubMed, EMBASE, Clinical Trials, Controlled Trials, The WHO International Clinical Trials Registry Platform, the Cochrane Central Register of Controlled Trials, CNKI, and Whipple Journal databases from January 1, 2016 to October 31, 2021. Two researchers independently screened the literature and extracted the data, and adopted a qualitative analysis method to evaluate the research status of the length and usage of screen time of school-aged students.

**Results:**

Fifty-three articles were included. Sixteen articles studied screen time length in the form of continuous variables. Thirty-seven articles studied screen time in the form of grouped variables. The average screen time of schoolchildren aged 6 to 14 was 2.77 h per day, and 46.4% of them had an average screen time ≥ 2 h per day. A growth trend could be roughly seen by comparing studies in the same countries and regions before and after the COVID-19 outbreak. The average rates of school-aged children who had screen time within the range of ≥ 2 h per day, were 41.3% and 59.4% respectively before and after January 2020. The main types of screen time before January 2020 were watching TV (20 literatures), using computers (16 literature), using mobile phones/tablets (4 literatures). The mainly uses of screens before January 2020 were entertainment (15 literatures), learning (5 literatures) and socializing (3 literatures). The types and mainly uses of screen time after January 2020 remained the same as the results before January 2020.

**Conclusions:**

Excessive screen time has become a common behavior among children and adolescents around the world. Intervention measures to control children's screen use should be explored in combination with different uses to reduce the proportion of non-essential uses.

## Background

Screen time refers to the time an individual spends using electronic or digital media devices such as televisions, smart phones, tablets or computers [1]. With the development of science and technology integrated into social life, smart devices such as mobile phones, computers and tablets are more and more widely used in work, study and daily life. Children are exposed to electronic products at a younger age and their screen time is increasing. Too much screen time can have negative effects on children's physical and mental health. First, the negative effect of screen time on eyesight has been confirmed in many countries’ studies [2, 3]. For example, the study by Hu Jia et al. showed that screen time ≥ 3 h per day (OR = 2.026, 95%CI:1.235 ~ 3.325) was a myopia risk factor for primary and middle school students [4]. Second, excessive screen time will also bring obesity, depression, sleep disorders and other health problems to children and adolescents [4–6].

The COVID-19 pandemic is still spreading across the globe, affecting the lives of billions of residents around the world. Various public institutions, including schools, have adopted a range of lockdown measures. More primary and middle schools have conducted online teaching, and the time for school-aged children to use electronic products for online learning has further increased. Diane Seguin et al. found that during the pandemic, the average daily screen time of Canadian children increased from over 2 h (2.6 h on average) to nearly 6 h (5.9 h on average)(t(73) = 9.04, *p* = 0.001). Screen time increased by a total of more than 3 h, and children's screen time increased further during the pandemic compared to pre-pandemic [7].

Due to the physical development stage of school-aged children, the effect of prolonged screen time on their physical and mental health is more obvious and irreversible than that of adults. The Physical Activity Guidelines for Chinese Children and Adolescents [8] released in 2017 states that, the screen time of Chinese children and adolescents should be limited to 2 h per day. Referring to the guidelines of the American Academy of Pediatrics [9], children under the age of 2 should not use electronic media, while the time of using it for children over 2 years old should be limited to 2 h per day. However, empirical studies on the actual length and use of current screen time of school-aged children are relatively scattered and insufficient. This study used the qualitative systematic review method to analyze the relevant studies on the length and use of screen time of school-aged children, in order to provide scientific basis for designing screen time interventions and perfecting the screen use guidelines for school-aged children.

## Methods

### Inclusion criteria

The types of literature include cross-sectional studies, cohort studies and case–control studies published in the form of peer-reviewed journal articles. The research subjects of the literature should include primary and secondary school students aged 6 to 14, including male and female. The literature published includes raw data, screen time values, age distribution, time distribution, and the screen use.

### Exclusion criteria

Unpublished, unoriginal and non-peer reviewed articles, case reports, letters or comments; the research subjects do not meet the age requirements (under 6 years old, over 14 years old); the literature does not describe screen use time in detail, lacks quantitative data and correlation verification, and is only empirical conclusion.

### The strategy of literature search

Search the literature in the public databases on PubMed, Clinical Trials, Controlled Trials, the WHO International Clinical Trials Registry Platform, EMBASE, the Cochrane Central Register of Controlled Trials, CNKI, and Whipple Journal. According to the phrases included the age group, and the screen use, "school-age child"/"primary school"/"junior high school student"/"primary and secondary school student"; "screen time"/" video time "/" electronic equipment "/" electronic products "/" multimedia equipment "/" digital equipment "are searched in the database. At the same time, search the references of the literature for other literature. The search time limit is from January 1, 2016 to October 31, 2021. The types of literature searched include cross-sectional studies, cohort studies and case–control studies. The search was limited to human studies reported either in English or in Chinese. All search phrases were modified according to MeSH terms.

### Literature screening and data extraction

According to the search strategy and inclusion and exclusion criteria, two researchers independently conduct literature screening. After the screening, the two researchers discuss the screening process and the inconsistent parts of the results to form a unified result. If no agreement were to reach, a third party should be consulted. The contents of the research extraction include: author, publishing time, research region, research type, sample characteristics, screen time length, use and influencing factors, research content and main results and conclusions.

### Risk evaluation and systematic evaluation of literature bias

The Cochrane risk assessment tool [10] is used to evaluate the literature quality of the included cross-sectional studies from the following aspects: random sequence generation, allocation hiding, blinding method, result data integrity, selective reporting and other biases. The bias risk has three possibilities: low risk, high risk and unknown bias risk. For observational studies, Newcastle–Ottawa Scale (NOS) [11] is used for quality assessment, which is scored from three parts: the selection of study population, comparability, exposure evaluation or result evaluation, and uses the semi-quantitative principle of star level system to evaluate literature quality. Studies with a score of 6 stars or more are defined as high quality and are included in this study. The quality assessment is conducted independently by the above-mentioned three researchers. In case of any dispute, a consensus shall be reached through discussion. In this study, Excel 2016 software was used to count the published literature, and qualitative analysis was performed on the included studies.

## Results

### Basic information and bias risk evaluation of included research

**Fig. 1:**
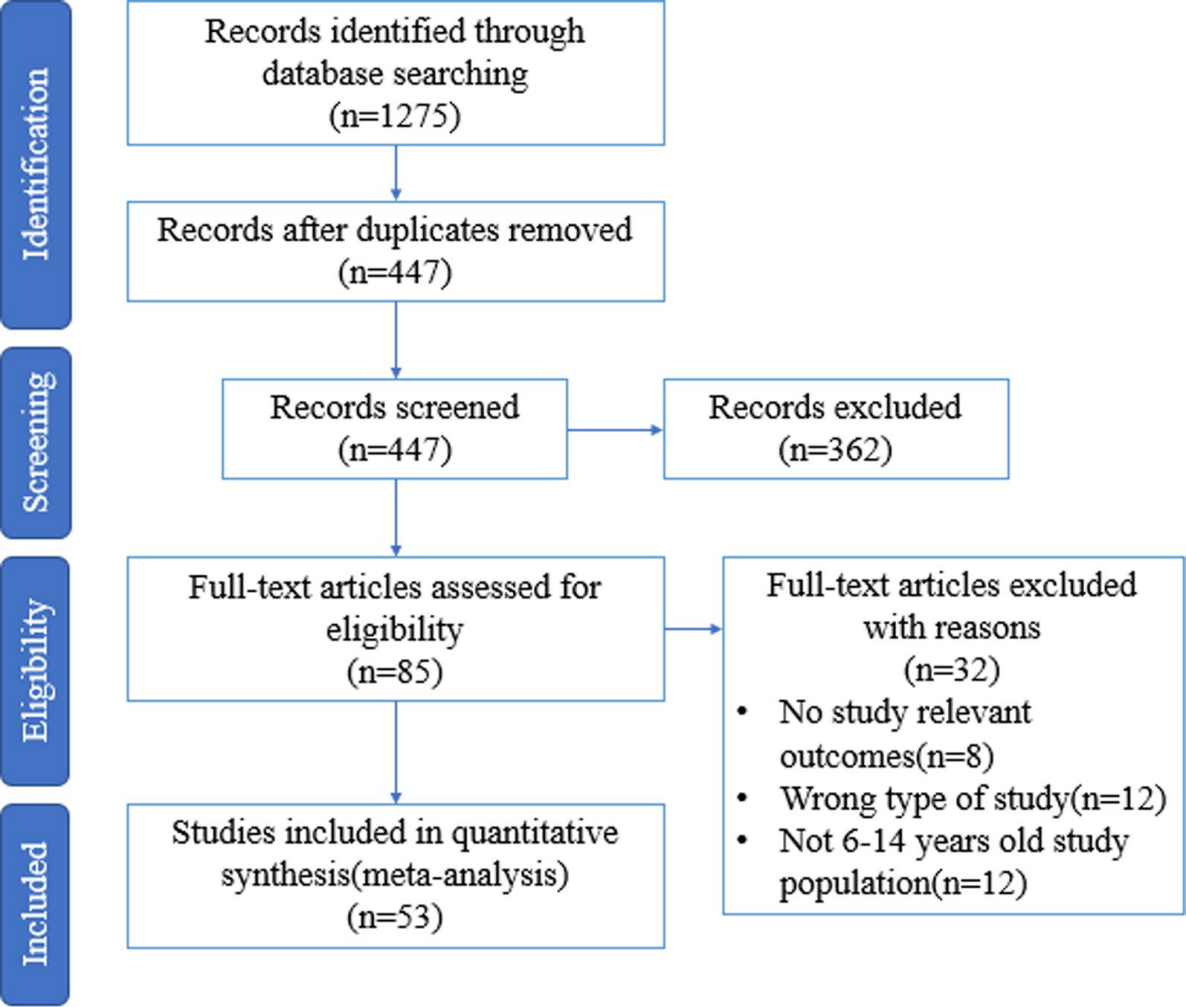
Flow chart of literature screening

**Fig. 2:**
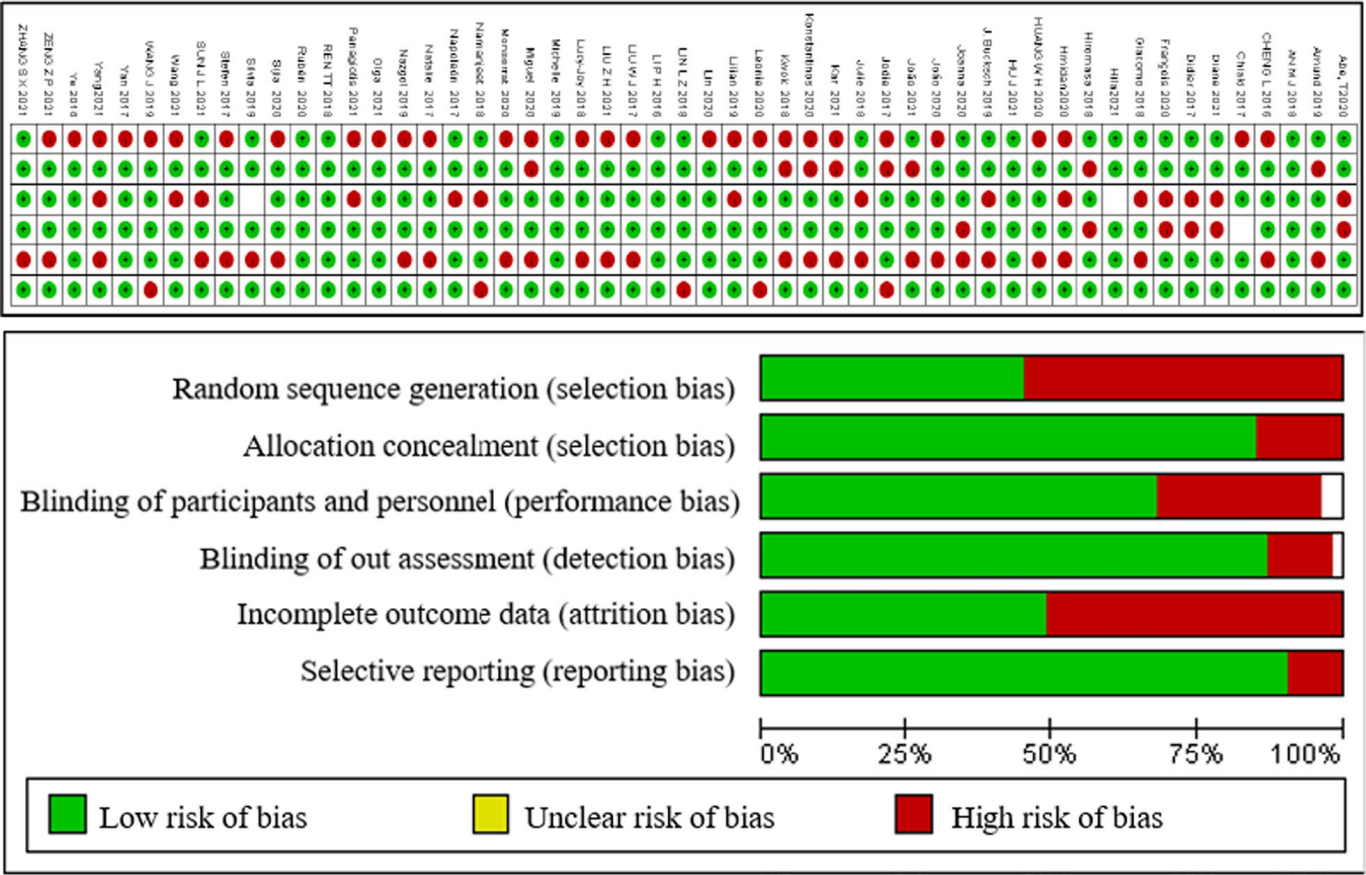
Bias risk evaluation results of different included studies (red indicators high risk, green indicators low risk)

The preliminary search obtained 1275 relevant literatures. After removing the duplicates and reading the literature titles and abstracts, through rounds of screening, two hundred and twenty-six literatures were excluded due to the lack of screen use data. Seventy-nine literatures were excluded due to inconsistent characteristics such as age and gender of the subjects. Thirty-six literatures were excluded due to inconsistent research types. Eight literatures were excluded due to incomplete content of the full text. Thirteen literatures were excluded because the research data source time was more than five years. Finally, fifty-three literatures [4–7, 12–60] were included. Their basic information was shown in Table 1. The literature screening process and results are shown in Fig. 1. Considering the representativeness of the sample population, we made unified screening regulations on the age of the study population, the difficulty in obtaining electronic devices, the family's economic ability, and the parents' education level of the study population. There were 19 Chinese literatures and 34 English literatures. In terms of research time, there were two literatures in 2016, eight literatures in 2017, ten literatures in 2018, seven literatures in 2019, thirteen literatures in 2020 and thirteen literatures in 2021. Nineteen literatures were from China (including Taiwan Province), 6 literatures from other Asian countries, 17 literatures from European countries, 9 literatures from American countries, 1 literature from African countries and 1 literature from Oceania countries. The screen time data in the literature were collected by questionnaire and database. There were 16 literatures with continuous screen time and 37 literatures with classified screen time. The evaluation results of the bias risk of different included studies are shown in Fig. 2.

**Table 1:** Basic features of the included literatures

Study	Publication year	Target area	Research design	Sample Size	Gender distribution	Age group (year)	Data measurement methods	Results of screen time related study	
								Length	Use
Hu et al. [4]	2021	China	Cross-sectional	882	m = 437, f = 445	6–18	Questionnaire	Average ST < 2 h/d accounted for 39.1%	
An et al. [5]	2018	China	Cross-sectional	2670	m = 1338, f = 1332	6–18	Questionnaire	Average ST of primary and middle school students in Beijing on school days ≤ 2 h/d accounted for 93.3%, and that at weekends accounted for 70.0%	
Liu et al. [6]	2021	China	Cross-sectional	1090	m = 561, f = 529	9–12	Questionnaire	Average ST of 8-year-olds ≤ 2 h/d accounted for 94.8%. The data for 9-year-olds was 94.2%, for 10-year-olds was 94.6%, and the overall study was 94.4%	
Diane et al. [7]	2021	Canada	Cross-sectional	73		6–12	Questionnaire	Average ST was 5.9 h/d	
Bel-Serrat et al. [12]	2019	19 European countries	Cross-sectional	1758	m = 908, f = 850	6–9	Database questionnaire	Northern Europe has the longest screen time (1.9 h/d), Eastern European countries have 1.7 h/d and Southern European/Mediterranean countries have the shortest screen time (1.4 h/d)	
Leonie et al. [13]	2020	Europe	Cross-sectional	2694	m = 1387, f = 1307	2–18	Database	Average ST was 13.2 h/w for children of 7–8 years old, 14.6 h/w for children of 9–10 years old, 18.4 h/w for children of 11 years old and above, and 13.7 h/w for the whole study population	
Miguel et al. [14]	2020	Spain	Cross-sectional	908		5–14	Database questionnaire	Average ST of children was 2.02 h/d (SD = 1.03)	
Didier et al. [15]	2017	Canada	Cross-sectional	1328	m = 674, f = 654	6–11	Database questionnaire	Children's average ST length was 2.3 h/d, using TV (1.6 h/d) and computer (0.7 h/d)	TV, games, learn, chat, emails, surf the Internet
Zhang et al. [16]	2021	China	Cross-sectional	5266		6–12	Questionnaire	Average ST of first grade ≥ 2 h/d on school days and at weekends accounted for 42.4% and 52.9%, 40.9% and 53.1% in second grade, 44.8% and 60.2% in third grade, 43.1% and 59.1% in fourth grade, 42.2% and 55.3% in fifth grade, and 39.6% and 50.4% in sixth grade	Online learning, TV, tablets/computers/mobile phones
Michelle et al. [17]	2019	The United States	Cross-sectional	11,875	m = 6188, f = 5681	9–10	Questionnaire	Children's ST: 1.26 h/d for TV/movies, 0.98 h/d for videos, 1.01 h/d for video games, 0.52 h/d for social media, 0.38 h/d for M-level games. 0.56 h/d for R-rated movies	TV/movies/ videos, video games, social media
Amund et al. [18]	2019	Norway	Cross-sectional	4509	m = 2128 ,f = 2381	11,13, 15,16	Questionnaire	The average ST was 6.1 h/d (SD4.3)	TV, games and other purposes
Julie et al. [19]	2018	Europe	Cross-sectional	10,696	m = 5380, f = 5589	5–13	Questionnaire	Children's average ST was 106.9 m/d, mainly for watching TV	
Rubén et al. [20]	2020	Spain	Cross-sectional	860	m = 437, f = 423	3–16	Questionnaire	Average ST was 2.0 h/d before home confinement, 4.9 h/d during confinement and 4.8 h/d after relaxation of confinement	Online games and learning
Olga et al. [21]	2021	Greece	Cross-sectional	1331	m = 600, f = 731	10–12	Questionnaire	Average ST was 1.52 h/d on weekdays and 3.19 h/d at weekends	TV, computer games
Nazgol et al. [22]	2019	Iran	Cross-sectional	23,043	m = 11,706, f = 11,337	6–18	Questionnaire	Average ST was 1.9 h/d (SD1.2), with 59.1% of subjects < 2 h and 40.9% of subject ≥ 2 h	TV, computer games
Joanna et al. [23]	2020	Poland	Cross-sectional	14,044	m = 6488, f = 7556	13–19	Questionnaire	Subjects ST (h/d): 13-year-old girls 2.5 ± 1.5 and boys 2.7 ± 1.6, 14-year-old girls 2.7 ± 1.5 and boys 3.0 ± 1.6	
Napoleón et al. [24]	2017	Spain	Cross-sectional	6487	m = 3269, f = 3218	6–9	Questionnaire	The average ST of subjects was 2.5 ± 1.4 h/d	
Jodie et al. [25]	2017	Canada	Cross-sectional	18,147		9–12	Questionnaire	The ST was 4.5 h/d for women and 5.2 h/d for men	
Chiaki et al. [26]	2017	Japan	Cross-sectional	426		6–12	Questionnaire	The children with an average ST length of 146 ± 80 m/d and > 2 h/d accounted for 59.8%	Games, TV, videos
Panagiotis et al. [27]	2021	Athens	Cross-sectional	91	m = 39, f = 52	8–12	Questionnaire	Average ST in three study groups was 2.3 h/d, 3.0 h/d, 2.7 h/d	
Ye et al. [28]	2018	China	Cross-sectional	1063	m = 510, f = 553	8–19	Questionnaire	Average ST on weekdays was 1.86 h/d for boys and 1.33 h/d for girls; 7.12 h/ d for boys and 5.86 h/d for girls at weekends	TV, mobile phones or tablets, computers for entertainment and learning purposes
Abe et al. [30]	2020	Japan	Cross-sectional	1794	m = 949, f = 845	9–15	Questionnaire	The average ST length ≥ 2 h/d accounted for 97.1%	
Namanjeet et al. [31]	2018	The United States	Cross-sectional	3421		6–11	Questionnaire	The average ST length ≥ 2 h/d accounted for 72.4%	TV and computers
Hmidan et al. [32]	2020	Arab	Cross-sectional	1023		9–12	Questionnaire	Average ST of watching TV/DVD/videos ≥ 2 h/d accounted for 34.8%; using computers ≥ 2 h/d accounted for 17.0%; playing video games ≥ 2 h/d accounted for 16.5%; using mobile electronic devices ≥ 2 h/d accounted for 21.5%	TV/DVD/Video, computers, video games, mobile electronic devices
Hila et al. [33]	2021	Israel	Cross-sectional	1758	m = 826, f = 932	11–17	Database questionnaire	The ST behavior of most children exceeded 2 h/d (higher-income families: 60.83%; non-high-income families: 63.91%)	TV and computers
Bucksch et al. [34]	2019	Germany, Poland, Slovenia, Czech Republic	Cross-sectional	18,781	m = 9295, f = 9486	11,13, 15	Database questionnaire	The total average ST length of the study population ≥ 3.5 h/d accounted for 67.7%. Average ST ≥ 3.5 h/d accounted for 69.4% in Czech Republic; 66.6% in Germany, 71.4% in Poland and 61.1% in Slovenia	TV and computers
Kar et al. [35]	2021	Australia	Cross-sectional	127	m = 54, f = 73	10–13	Database questionnaire	Average ST ≥ 2 h/d accounted for 67.7%	Entertainment purposes
Gallant et al. [36]	2020	Canada	Cross-sectional	923	m = 416, f = 507	8–19	Database questionnaire	Average ST of girls ≥ 2 h/d accounted for 42.5%, and average ST of boys ≥ 2 h/d accounted for 42.3%	
Guo et al. [37]	2021	China	Cross-sectional	10,416	m = 5219 f = 5197	10–16	Online questionnaire	During the COVID-19 pandemic, average ST less than 2 h/d accounted for 31.6%	Learning and entertainment purposes
Schmidt et al. [38]	2017	Canada	Cross-sectional	18,147	m = 9243, f = 8904	9–12	Questionnaire	Average ST of 6 to 10-year-olds < 2 h/d accounted for 79.7%, and that of 11 to 13-year-olds accounted for 40.1%	
Lilian et al. [39]	2019	Berlin	Cross-sectional	2122		12–13	Questionnaire	Average ST less than 2 h/d accounted for 81.5%	TV and computers
Giacomo et al. [40]	2018	Italy	Cross-sectional	3291		11–15	Questionnaire	Average ST of watching TV ≥ 2/d accounted for 46.4%; playing games ≥ 2/d accounted for 29.2%; using computers ≥ 2/d accounted for 38.8%	TV, games, computers
Lin et al. [41]	2020	Taiwan, China	Cross-sectional	1005	m = 503, f = 502	6–13	Questionnaire	Average ST > 2 h/d accounted for 54.9%	Computers and TV
Kwok et al. [42]	2018	Europe	Cross-sectional	61,329	m = 27,832, f = 33,497	11,13, 15	Database	Average ST length of boys > 2 h/d on weekdays accounted for 62% and that among girls > 2 h/d accounted for 59%. Average ST of boys at weekends > 2 h/d accounted for 79% and that among girls > 2 h/d accounted for 77%	computer games and TV
Natalie et al. [43]	2017	UK	Cross-sectional	527	m = 253, f = 274	11–12	Questionnaire	Average ST > 2 h/d accounted for 69.7%	TV/DVD
Monserrat et al. [44]	2020	Spain	Cross-sectional	402	m = 216, f = 186	2–14	Questionnaire	Average ST ≥ 2 h/d among children aged 6–14 accounted for 60.6%	TV and games
Silveira et al. [45]	2020	Italy	Cross-sectional	1200	m = 545, f = 655	6–17	Questionnaire	Average ST of children (34.3%) and adolescents (48.2%) was ≥ 2 h/d	
Souza et al. [46]	2021	Brazil	Cross-sectional	1438	m = 675, f = 763	10–14	Questionnaire	Average ST > 2 h/d accounted for 59.6%	Computer, TV and games
Konstantinos et al. [47]	2020	Norway	Cross-sectional	177,091	m = 90,821, f = 86,270	8–17	Database questionnaire	Average ST ≤ 2 h/d accounted for 65.4%	Computer, non-learning purposes, mobile phones, TV, games
Hiromasa et al. [48]	2018	Japan	Cross-sectional	1374	m = 679, f = 695	6–15	Questionnaire	Average ST of watching TV among boys < 2 h accounted for 58.6% and that among girls accounted for 56.6%	TV, computer and mobile phone
Lucy-Joy et al. [49]	2018	Kenya	Cross-sectional	563	m = 262, f = 301	9–11	Database questionnaire	Average ST < 2 h/d on school days accounted for 67.9% and average ST < 2 h/d at weekends accounted for 25.8%	
Wang et al. [50]	2020	China	Cross-sectional	111,173		10–14	Database questionnaire	Average ST < 2 h/d on school days accounted for 66.8% and the average ST length < 2 h/d at weekends accounted for 38.4%	
Yan et al. [51]	2017	China	Cross-sectional	2625		7–12	Database questionnaire	The average ST length > 14 h/w accounted for 35.43%	TV, video games, social networking sites, videos, search for news and learning materials
Zeng et al. [52]	2021	China	Cross-sectional	16,545	m = 8344, f = 8201	13–22	Questionnaire	Average ST ≤ 2 h/d accounted for 81.6% that of boys accounted for 80.9% and that of girls accounted for 82.4%	
Cheng et al. [53]	2016	China	Cross-sectional	1170		7–11	Questionnaire	Average ST ≤ 2 h/d accounted for 85.7%	
Huang et al. [54]	2020	China	Cross-sectional	12,357	m = 6292, f = 6065	12	Questionnaire	Average ST ≥ 2 h/d accounted for 18.9%, with that of boys accounted for 18.5% and girls accounted for 19.1%	
Lin et al. [55]	2018	China	Cross-sectional	1889		7–11	Questionnaire	Average ST ≥ 2 h/d accounted for 22.9%	
Liu et al. [56]	2017	China	Cross-sectional	2859		7–12	Questionnaire	Average ST ≥ 2 h/d on school days accounted for 17.7% and at weekends accounted for 33.6%	
Ren et al. [57]	2018	China	Cross-sectional	2644	m = 1402, f = 1242	7–18	Questionnaire	Average ST of Kashgar Uyghur children < 2 h/d accounted for 50.1%. Average ST of primary school < 2 h/d accounted for 60.2% and that of middle school accounted for 56.2%. The overall weekday ST length < 2 h/d accounted for 93.3%, and at weekends accounted for 69.7%	
Sun et al. [58]	2021	China	Cross-sectional	1432	m = 758, f = 674	6–16	Questionnaire	Average ST ≥ 2 h/d accounted for 44.5%	
Wang et al. [59]	2019	China	Cross-sectional	1062	m = 576, f = 486	11–19	Questionnaire	Average ST ≥ 2 h/d accounted for 20.3%	
Wang et al. [60]	2021	China	Cross-sectional	1585	m = 811, f = 774	6–14	Questionnaire	Average ST > 2 h/d accounted for 57.5% among 6–7 years old, 57.1% among 8–10 years old, and 47.6% in middle school (11–14 years)	Computer, tablets and mobile phones

### Average daily length of screen time among schoolchildren aged 6–14 (continuous variable)

**Fig. 3:**
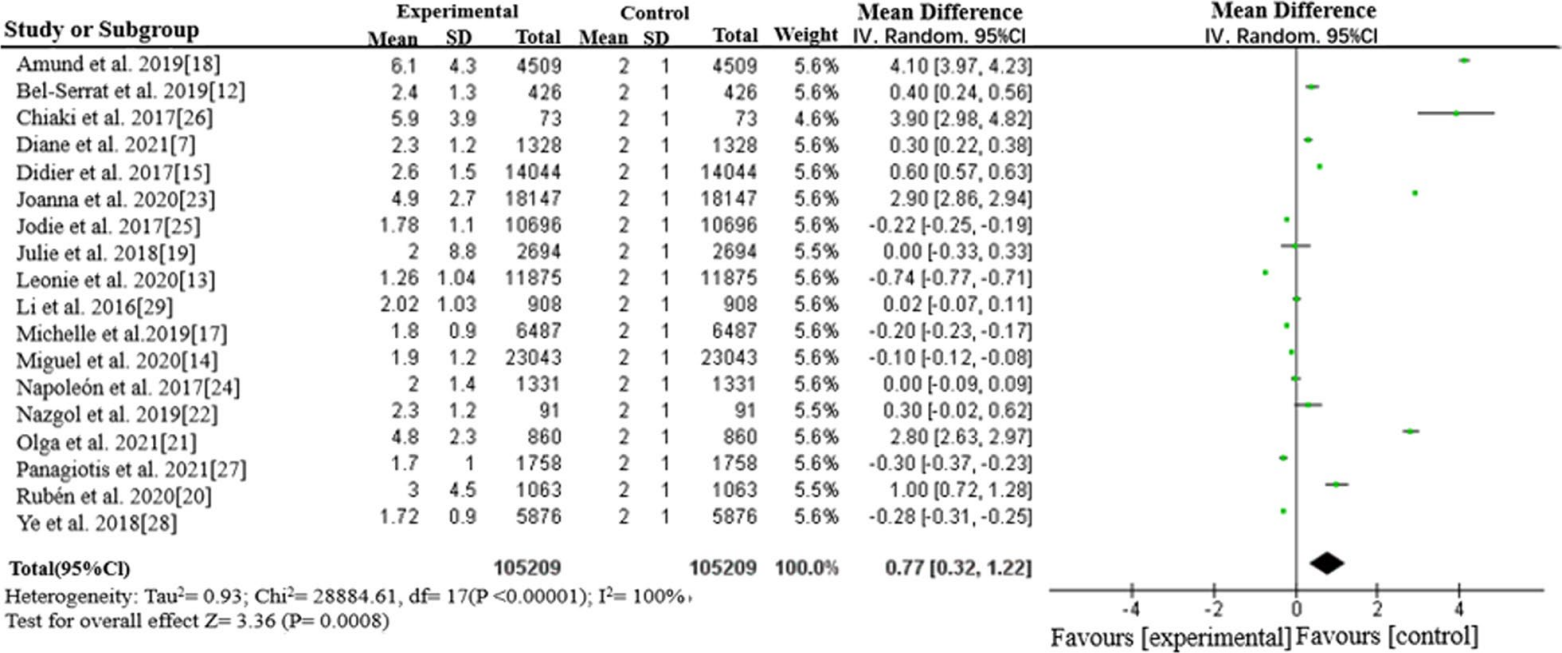
Forest plot for screen time of 6–14 year old school children (continuous variable)

In 55 literatures, sixteen of them studied screen time length in the form of continuous variables. Sixteen literatures investigated the average daily length and standard deviation of the group by screen time and other health behavior factors. A total of 105,209 primary and middle school students aged 6 to 14 years were included in the study. Taking the international recommended length of screen time—2 h per day as the control parameter, the average length and standard deviation of the screen time of each literature were entered. Meta-analysis carried out by RevMan software showed that the average screen time of the included literature was + 0.77 h higher than the control parameter and the average screen time was 2.77 h per day (95% CI: 0.32 ~ 1.22).The analysis results are shown in Fig. 3.

### Average daily length of screen time for Schoolchildren aged 6–14 (Classification variable)

**Fig. 4:**
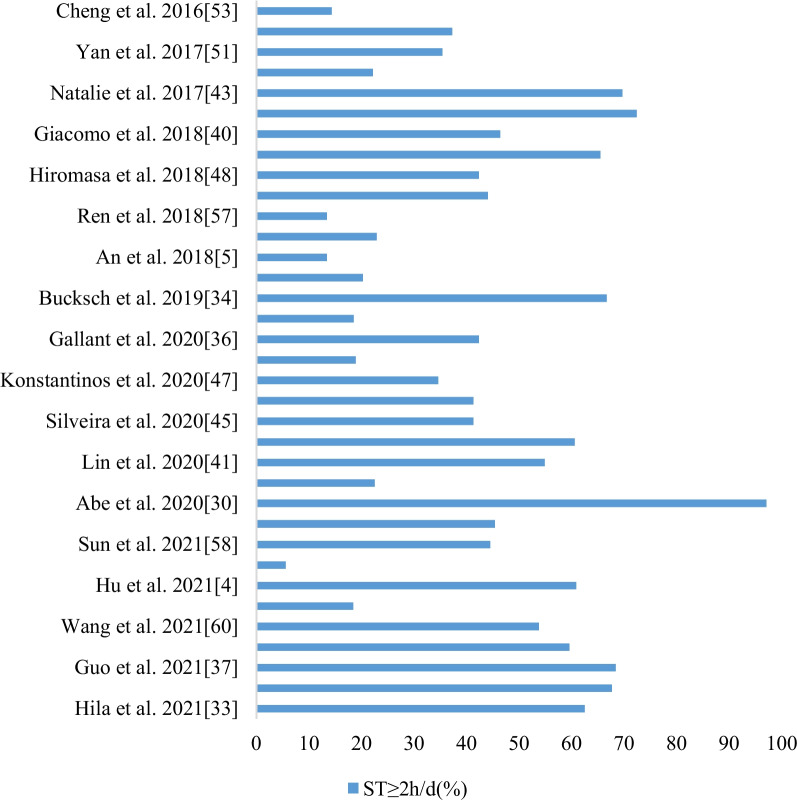
Screen time of 6–14 year old school children (classification variable)

Among the 55 literatures, thirty-seven expressed screen time in the form of grouped variables. Screen time < 2 h per day and ≥ 2 h per day were defined as screen time in 35 of the 37 classification variable literatures. Two literatures that only provided data on screen time use were not included in the bar chart. Among the included literatures published in 2021, there were four papers whose actual data collection took place in 2021, while the rest of the literatures published in 2021 reported data was collected in 2020 and before. A total of 472,042 primary and middle school students aged 6 to 14 years were included in the study. With the included literatures presented in chronological order, the bar chart showed the proportion of groups with average screen time ≥ 2 h per day in the whole study population. The results showed that 46.4% of primary and middle school students aged 6 to 14 years had screen time within the range of ≥ 2 h per day. A growth trend could be roughly seen by comparing studies in the same countries and regions before and after the COVID-19 outbreak. The average rates of school-aged children, who had screen time within the range of ≥ 2 h per day, were 41.3% and 59.4% respectively before and after January 2020. The statistical results are shown in Fig. 4.

### Main uses of screen time for school-aged children

In the included literatures, twenty-five analyzed the types and uses of screen time among schoolchildren aged 6 to 14. The full text of the literature were read to get the classification of the screen devices, including televisions, mobile phones, tablets and computers. The classification of screen use were put into three categories, namely, learning, entertainment (including watching video and video games) and social interaction. The number of literatures and samples for each kind of use were counted. A total of 330,119 schoolchildren aged 6 to 14 were included in this indicator. Calculated according to the statistical sequence of the sample size of the literature study, the results showed that the main types of screen time before January 2020 were watching TV (20 literatures), using computers (16 literature), using mobile phones/tablets (4 literatures). The mainly uses of screens before January 2020 were entertainment (15 literatures), learning (5 literatures) and socializing (3 literatures). The types and mainly uses of screen time after January 2020 remained the same as the results before January 2020, as shown in Table 2.

**Table 2:** Main uses of school-age children’s screen time

Study	Publication year	Screen type			Screen time purpose		
		TV	Computer	Phone/Pad	Study	Entertainment	Social
Didier et al. [15]	2017	Y			Y	Y	Y
Zhang et al. [16]	2021	Y	Y	Y	Y		
Michelle et al. [17]	2019	Y				Y	Y
Amund et al. [18]	2019	Y				Y	
Rubén et al. [20]	2020				Y	Y	
Olga et al. [21]	2021	Y				Y	
Nazgol et al. [22]	2019	Y	Y			Y	
Chiaki et al. [26]	2017	Y				Y	
Ye et al. [28]	2018		Y		Y		
Li et al. [29]	2016	Y	Y			Y	
Namanjeet et al. [31]	2018	Y	Y				
Hmidan et al. [32]	2020	Y	Y	Y		Y	
Hila et al. [33]	2021	Y	Y				
Bucksch. et al. [34]	2019	Y	Y				
Lilian et al. [39]	2019	Y	Y				
Giacomo et al. [40]	2018	Y	Y			Y	
Lin et al. [41]	2020	Y	Y				
Kwok et al. [42]	2018	Y	Y			Y	
Natalie et al. [43]	2017	Y					
Monserrat et al. [44]	2020	Y				Y	
João et al. [46]	2021	Y	Y			Y	
Konstantinos et al. [47]	2020	Y	Y	Y		Y	
Hiromasa et al. [48]	2018		Y	Y			
Yan et al. [51]	2017	Y	Y		Y	Y	Y
Wang et al. [60]	2021		Y	Y			
Total		21	17	5	5	15	3

## Discussion

From smartphones and social media to TV and tablet-based online courses, today’s school-aged children are constantly inundated by technology. The primary purpose of this review was to summarize the current situation of length and use of screen time of school-aged children. Our findings show that excessive screen time among schoolchildren aged 6–14 is very common and has become a serious public health problem in high—and middle-income countries. Excessive screen time has a variety of effects on the health of school-aged children, including emotional, sleep, behavioral problems, and affects the growth and cognitive development of school-aged children. Some high-income countries, such as the United States [61] and Germany [62], have developed guidelines for restrictions on digital media overuse across age groups, while some low—and middle-income countries have not developed such screen time guidelines. In 2021, the National Health Commission issued Appropriate Technical Guidelines for Prevention and control of Myopia in Children and Adolescents (updated version) [63], which suggested that families should "not put TV and other video products in children's bedrooms", but did not put forward suggestions on screen duration. This review might be useful for the policymakers in formulating or refining guidelines for limiting the excessive digital-media usage for school-aged groups in these countries.

Instead of school settings, home-based television viewing and home-based computers are two primary types of screen viewing of school-aged children. The home setting, especially parents, plays a vital role in deciding the type and length of screen viewing. Parents’ attitudes, beliefs, norms, and behaviors shape and create a shared social and physical environment in the home setting, and this environment affects children’s possibilities for different types of behaviors [64]. Higher parental self-efficacy to limit screen time is associated with less children’s screen time, whereas availability of media equipment is associated with increased children’s screen time [65]. Therefore, health promotion programs are needed to help raise parents' awareness and ability to help reduce children’s excessive screen time. Among different purposes of screen time for school-aged children, the main purpose is spent on entertainment rather than learning, which offers the possibility of reducing long screen time. Parents could set time limits on the use of entertainment software on electronic devices, or replace screen use with outdoor activities. It is also relevant to study further the screen use preferences of students of different ages, and to distinguish the use time of different screen media such as TV, computer and mobile phone. This knowledge would be valuable for the development of effective interventions aiming to diminish the school-aged children’s screen time.

During disease pandemic such as COVID-19, screen usage may become more prevalent through periods of school closures, lockdowns, social isolation, and online learning classes. Public health policies and health promotion strategies targeting parents are needed to raise awareness of the adverse health effects associated with excessive screen time [66]. From our findings, comparing the literature data before 2020 with those after 2020, the increase in screen time of primary and middle school students in the same countries and regions is obvious. There are also relevant studies [67] that due to the impact of the epidemic, the proportion of children whose screen time of electronic products was longer than 3 h per day rose from 9.16% before the epidemic to 19.20% after the epidemic. When literatures were searched, the publication years of literature included the time of epidemic. Compared with those before 2019, there has been a significant increase in screen time reported in the literature since 2020, which is related to the fact that the children have been forced to stay at home longer, and online teaching has led to increased average exposure to electronic devices during the pandemic. Since the online learning is “required” by schools, it raises a triple dilemma among maintaining school-learning, prevention of communicable diseases, and reducing excessive screen time, which needs further discussion. In addition, healthcare workers could provide health education and health consulting service on appropriate screen use behavior, how to improve digital media environment at home, and raise awareness of adverse health effects of screen time. Fitness and entertainment facilities shall be provided at the community level to reduce screen time, and enhance the physical activity level of children and adolescents. An integration of family, community, school, and health systems should be considered to design for intervention model of screen time behaviors.

This study has some limitations. First, according to the research types included in the literature, this study selected the international mainstream methodological quality scale for quality evaluation, but the quality of the relevant original research methodology was limited and not rigorous. It may have reduced the credibility of the conclusions. Second, in the included studies, national conditions and medical systems vary from country to country. The included literatures mainly focus on the health effects of screen time. The standards of screen time data collection and classification were not uniform among studies, which made the statistical results may deviate from the actual situation. In addition, the age range of some study subject included in the literature is not completely in the age range of 6–14 years old. Although only the data of the study subjects in accordance with the age group were selected in the data analysis, there were cases where a single data represented the level of the entire age group, and the sample size of the study subjects of each age group was not balanced, which may cause some bias to the conclusion. Only published literatures were searched, which may lead to incomplete data acquisition and potential publication bias. Third, because of the exclusion of literature published in languages other than English and Chinese, the research results were not representative in these language regions. Last, seventeen of the included literature were published after January 2020, but their data was collected before January 2020. New papers investigating screen time during COVID-19 pandemic have been published after our target date. Those latest data collection could be continued in the future to fully reflect the impact of the pandemic on screen time.

## Conclusions

Focusing on school-aged children, this study systematically assessed the specific length and main uses of screen time in school-aged children aged 6–14, providing a baseline reference level for excessive screen time in school-aged children. It also provides ideas for interventions to reduce long screen time. However, the quality of the existing research is uneven, and the research types and quantity are relatively scarce. Further empirical research is needed to confirm the above conclusions.

## Data Availability

The datasets during and/or analysed during the current study available from the corresponding author on reasonable request.

## Abbreviation

ST: Screen time: time spent using the computer, watching TV, playing video games and other multimedia screens
